# Climate-induced shifts in sulfate dynamics regulate anaerobic methane oxidation in a coastal wetland

**DOI:** 10.1126/sciadv.ads6093

**Published:** 2025-04-23

**Authors:** Jaehyun Lee, Yerang Yang, Hojeong Kang, Genevieve L. Noyce, J. Patrick Megonigal

**Affiliations:** ^1^ Climate and Environmental Research Institute, Korea Institute of Science and Technology, Seoul, Republic of Korea.; ^2^ Smithsonian Environmental Research Center, Edgewater, MD, USA.; ^3^ School of Civil and Environmental Engineering, Yonsei University, Seoul, Republic of Korea.

## Abstract

Anaerobic methane oxidation (AMO) is a key microbial pathway that mitigates methane emissions in coastal wetlands, but the response of AMO to changing global climate remains poorly understood. Here, we assessed the response of AMO to climate change in a brackish coastal wetland using a 5-year field manipulation of warming and elevated carbon dioxide (*e*CO_2_). Sulfate (SO_4_^2−^)–dependent AMO (S-DAMO) was the predominant AMO process at our study site due to tidal inputs of SO_4_^2−^. However, SO_4_^2−^ dynamics responded differently to the treatments; warming reduced SO_4_^2−^ concentration by enhancing SO_4_^2−^ reduction, while *e*CO_2_ increased SO_4_^2−^ concentration by enhancing SO_4_^2−^ regeneration. S-DAMO rates mirrored these trends, with warming decreasing S-DAMO rates and *e*CO_2_ stimulating them. These findings underscore the potential of climate change to alter soil AMO activities through changing SO_4_^2−^ dynamics, highlighting the need to incorporate these processes in predictive models for more accurate representations of coastal wetland methane dynamics.

## Teaser

Climate change affects sulfate-dependent anaerobic methane oxidation by shifting sulfur cycling in a coastal wetland.

## INTRODUCTION

Coastal wetlands have been traditionally considered a minor source of methane (CH_4_) due to the presence of sulfate (SO_4_^2−^), which can outcompete methanogenic communities for electron donors (*1*, *2*). However, recent studies have shown that CH_4_ emissions in coastal wetlands are highly variable and can often exceed carbon sequestration in terms of CO_2_ equivalents (*3*–*6*). Despite the significance of CH_4_ emissions in the carbon budget of coastal wetlands (*7*), a considerable degree of uncertainty persists in accurately quantifying their CH_4_ emissions (*8*, *9*). Moreover, the increasing threat of climate change presents a substantial challenge to the fragile equilibrium of these ecosystems (*10*, *11*). Warming temperatures, sea level rise, and elevated atmospheric CO_2_ are among the numerous climate-induced changes that influence the resilience and greenhouse gas balance of coastal wetlands (*10*, *12*, *13*). These changes often disrupt microbial processes that regulate CH_4_ dynamics (*14*–*16*). In this context, a comprehensive understanding of the CH_4_ dynamics in coastal wetlands becomes essential for predicting and managing future contributions to the global CH_4_ budget.

CH_4_ dynamics in ecosystems are primarily governed by two opposing biogeochemical processes, CH_4_ production mediated by methanogens and its subsequent oxidation facilitated by methanotrophs (*17*). CH_4_ oxidation serves as a critical control mechanism, mitigating up to 90% of the CH_4_ produced under anoxic conditions in soils before its atmospheric dispersion (*18*, *19*). Oxidation processes vary by the availability of terminal electron acceptors: aerobic oxidation relies on oxygen (O_2_), while anaerobic oxidation uses SO_4_^2−^ [SO_4_^2−^-dependent anaerobic CH_4_ oxidation (S-DAMO)], nitrate (NO_3_^−^)/nitrite (NO_2_^−^) [NO_3_^−^/NO_2_^−^-dependent anaerobic CH_4_ oxidation (N-DAMO)], manganese/iron oxides, or oxidized humic substances (*20*). In environments abundant in O_2_, the aerobic pathway dominates because of the superior free energy yield of O_2_ (*20*). However, in O_2_-deprived systems characteristic of coastal zones, rivers, and lakes, anaerobic pathways assume a dominant role in the CH_4_ cycle. Anaerobic CH_4_ oxidation (AMO) consumes a median of 71% of the CH_4_ produced in the anoxic zones of inundated environments such as coastal ecosystems, wetlands, paddy systems, lakes, and rivers, where substantial amounts of CH_4_ are emitted into the atmosphere (*21*).

S-DAMO is performed by anaerobic methanotrophic (ANME) archaea, represented by three different phylogenic clusters (ANME-1, ANME-2, and ANME-3) (*21*). ANME-1 and ANME-2 are the most abundant group of ANME archaea, which are widely distributed in anoxic environments, while ANME-3 archaea mostly exist in submarine mud volcanoes or marine CH_4_ seeps (*22*, *23*). S-DAMO activity is typically observed in marine environments (marine sediment, coastal wetland soil, etc.) and is the dominant AMO pathway in these environments because SO_4_^2−^ is the major redox-active compound in seawater (*21*). A previous study estimated that S-DAMO removes 71 to 96% of CH_4_ produced by methanogens in a coastal wetland complex with varying salinities (1.4 to 34.5) (*24*), fractions that are similar to the limited number of studies on unvegetated marine ecosystems (*22*). However, S-DAMO can also play an important role in CH_4_ cycling in freshwater environments where SO_4_^2−^ is commonly depleted (*25*).

N-DAMO is a more recently found pathway of AMO (*26*, *27*). It plays a crucial role in the biogeochemical cycles of carbon and nitrogen. It couples CH_4_ oxidation with the reduction of NO_2_^−^ or NO_3_^−^ (*28*, *29*) and also facilitates ammonium oxidation linked to the oxidation of organic matter (*30*). Two microbes have been identified through enrichment cultures: *Methylomirabilis oxyfera*, which was identified in freshwater sediment and couples AMO to NO_2_^−^ reduction (*26*), and *Methanoperedens nitroreducens*, which was identified in both freshwater sediment and wastewater sludge, couples NO_3_^−^ reduction to AMO (*31*).

In soil environments, various methanotrophs compete for the limited carbon source, CH_4_, using different electron acceptors including O_2_, NO_2_^−^/NO_3_^−^, and SO_4_^2−^. Methanotrophic pathways with higher free energy yield are more likely to outcompete others for CH_4_ utilization (*20*). In an anoxic environment where NO_2_^−^/NO_3_^−^ and SO_4_^2−^ coexist, N-DAMO is more likely to occur than S-DAMO, as NO_2_^−^/NO_3_^−^ is more thermodynamically favored electron acceptors than SO_4_^2−^ (*20*, *32*). Therefore, the N-DAMO process is mainly found in freshwater environments such as terrestrial wetlands, rivers, and lakes and is the dominant AMO pathway in these environments (*27*, *33*–*35*). Meanwhile, in coastal ecosystems with an abundant supply of SO_4_^2−^ from seawater, S-DAMO is more likely to be the dominant AMO process (*21*). An exception is coastal ecosystems where anthropogenic NO_2_^−^/NO_3_^−^ inputs from rivers support N-DAMO as a more dominant AMO process than S-DAMO. For example, a previous study assessed S-DAMO and N-DAMO separately in the intertidal zone of the East China Sea and found that S-DAMO accounted for 35% of AMO, with the remainder attributed to N-DAMO activity (*36*). It was also suggested that N-DAMO is the dominant AMO pathway in intertidal flats of China and plays a key role in the nitrogen cycle (*37*–*39*).

Climate change phenomena such as warming temperatures and elevated levels of atmospheric CO_2_ may substantially influence the rate of AMO in coastal wetlands by changing the cycling and availability of key electron acceptors. For instance, a previous study revealed that warming markedly enhances SO_4_^2−^ depletion by increasing SO_4_^2−^ reduction rates (*15*). These changes in SO_4_^2−^ dynamics by warming can consequently affect S-DAMO activity. Moreover, warming can reduce the total nitrogen concentration in the soil by accelerating denitrification and the nitrogen turnover rate, as shown in a coastal wetland in the Yangtze Estuary (*40*), potentially affecting N-DAMO activity. Meanwhile, elevated CO_2_ (*e*CO_2_) has been shown to enhance root growth in coastal wetlands, thereby enhancing O_2_ transport belowground (*16*). This process could potentially decrease AMO activity through competition with aerobic methanotrophs or enhance AMO by regenerating NO_2_^−^/NO_3_^−^ and SO_4_^2−^ as AMO substrates. These changes in electron acceptor dynamics driven by climate change could substantially alter AMO rates and, subsequently, CH_4_ emissions from coastal wetland ecosystems.

While prior research has elucidated the effects of climate change on AMO and methanogenesis (*19*, *41*–*43*), our understanding of its influence on AMO remains limited. Despite this gap in knowledge, it is established that AMO substantially influences coastal wetland CH_4_ dynamics, removing up to 90% of CH_4_ produced by methanogens (*22*, *24*). Therefore, understanding how AMO responds to climate change and the mechanisms driving this response is essential for accurately predicting future CH_4_ dynamics in a changing climate (*44*). Here, we used an in situ climate change manipulation experiment in a coastal wetland to elucidate the response of AMO to warming temperatures and *e*CO_2_. We hypothesized that (i) S-DAMO is the dominant AMO pathway at our study site given the limited availability of NO_2_^−^/NO_3_^−^; (ii) warming reduces AMO activity due to warming-induced SO_4_^2−^ depletion; and (iii) *e*CO_2_ enhances the transport of O_2_ belowground, allowing aerobic methanotrophs to outcompete AMO microbes, which, in turn, decreases AMO activity.

## RESULTS

The study was conducted at the Smithsonian’s Global Change Research Wetland (GCReW), a brackish high marsh on the Chesapeake Bay, USA (38°55′N, 76°33′W), using the Salt Marsh Accretion Response to Temperature Experiment (SMARTX) (*45*). Four climate treatments were used to investigate the response of AMO activity and other biogeochemical characteristics to warming temperature and elevated atmospheric CO_2_ concentration. Whole-ecosystem temperature (plant canopy and soil to 1.5 m deep) was maintained at ambient levels, which varied naturally, and was instantaneously warmed to +5.1°C above ambient. Atmospheric CO_2_ was maintained at ambient concentration and at 350–part per million (ppm) CO_2_ above ambient concentration. The four treatments were fully crossed yielding (i) ambient (ambient temperature and CO_2_), (ii) *e*CO_2_ (ambient temperature and elevated CO_2_), (iii) +5.1°C (+5.1°C warming above ambient temperature), and (iv) +5.1°C + *e*CO_2_ (both warming and elevated CO_2_). This full design was applied in a plant community dominated by a C_3_ sedge, while a subset of the design involving only the temperature treatments was applied in a plant community dominated by C_4_ grasses.

### Effect of climate change on SO_4_^2−^ concentration, redox potential, and fine-root productivity

**Fig. 1.**
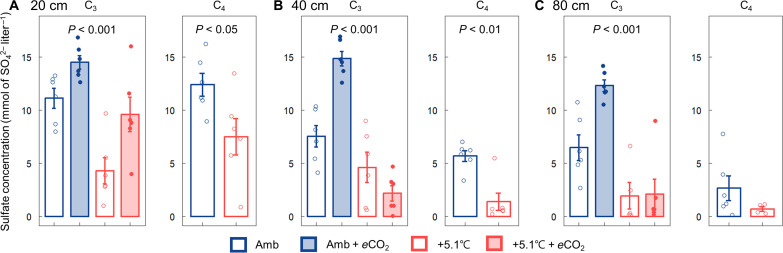
Porewater SO_4_^2−^ concentration in ambient and warmed plots with and without *e*CO_2_ across the different depths. Porewater SO_4_^2−^ concentration measured in May and July 2022 at depths of (**A**) 20 cm, (**B**) 40 cm, and (**C**) 80 cm in two warming treatments (ambient and ambient + 5.1°C) crossed with *e*CO_2_ (ambient and ambient + 350-ppm CO_2_) in C_3_ and C_4_ communities. A linear mixed-effects model and Mann-Whitney *U* test were used to test the difference in SO_4_^2−^ concentration between the treatments in C_3_ and C_4_ communities, respectively. Error bars indicate the SEM (*n* = 6). Amb, ambient.

Overall, warming led to decreased porewater SO_4_^2−^ concentration, whereas the influence of *e*CO_2_ was depth dependent (Fig. 1). In the C_3_ community, warming markedly decreased SO_4_^2−^ concentration at all measured depths (20, 40, and 80 cm) compared to the ambient conditions. The plots exposed to only *e*CO_2_ showed increased SO_4_^2−^ concentration at all measured depths, but this effect was absent in the deeper layers (40 and 80 cm) when crossed with the warming treatment. In the C_4_ community, warming led to a significant decrease in SO_4_^2−^ concentration at depths of 20 cm (Fig. 1A, *P* < 0.05) and 40 cm (Fig. 1B, *P* < 0.01), but the difference in SO_4_^2−^ concentration between the warming plots and the ambient plots at 80 cm was more subtle (Fig. 1C). The overall trend of SO_4_^2−^ concentration at 20 cm observed from 2017 to 2022 follows a trend similar to that in 2022, where warming led to a reduction in SO_4_^2−^ concentration and *e*CO_2_ increased it (fig. S1). SO_4_^2−^ depletion exhibited an opposite trend to SO_4_^2−^ concentration, with warming increasing SO_4_^2−^ depletion, while *e*CO_2_ decreased it (fig. S2).

**Fig. 2.**
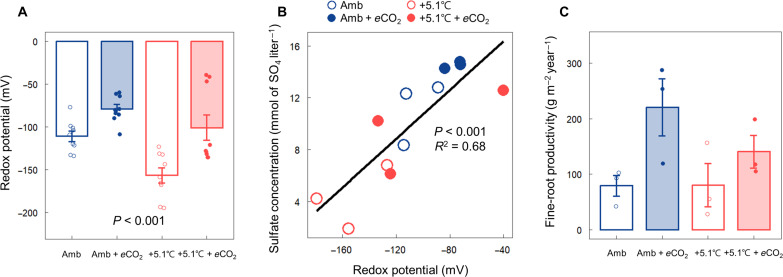
Porewater redox potential and fine-root productivity in ambient and warmed plots with and without *e*CO_2_ in the C_3_ community. (**A**) Monthly average redox potential measured at a depth of 20 cm from May to July 2022 in two warming treatments (ambient and ambient + 5.1°C) crossed with *e*CO_2_ (ambient and ambient + 350-ppm CO_2_) in the C_3_ community. (**B**) A positive correlation between redox potential and SO_4_^2−^ concentration measured at a depth of 20 cm in the C_3_ community. (**C**) Fine-root productivity (0 to 40 cm in depth) from November 2021 to November 2022. A linear mixed-effects model and one-way analysis of variance (ANOVA) were used to test the difference in redox potential and fine-root productivity, respectively, between the treatments. Error bars indicate the SEM [*n* = 6 for (A) and *n* = 3 for (C)].

Redox potential at a depth of 20 cm mirrored the treatment effects on SO_4_^2−^ concentration in the C_3_ community (redox was not measured in the C_4_ community). *e*CO_2_ increased redox potential, while warming decreased it (Fig. 2A, *P* < 0.001). In addition, the redox potential showed a significant positive correlation with SO_4_^2−^ concentration [Fig. 2B, coefficient of determination (*R*^2^) = 0.68, *P* < 0.001]. *e*CO_2_ increased fine-root productivity, while warming showed no effect in the C_3_ community in 2022 (Fig. 2C).

### Response of AMO process to climate change

**Fig. 3.**
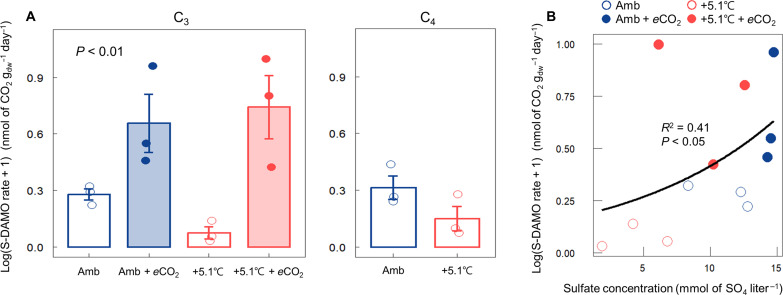
Differences in S-DAMO rate in ambient and warmed plots with and without *e*CO_2_. (**A**) S-DAMO rates in two warming treatments (ambient and ambient + 5.1°C) crossed with *e*CO_2_ (ambient and ambient + 350-ppm CO_2_) in C_3_ and C_4_ communities. (**B**) A positive correlation between S-DAMO rate and SO_4_^2−^ concentration in the C_3_ community. A one-way ANOVA and Mann-Whitney *U* test were used to test the difference in S-DAMO rate between the treatments in C_3_ and C_4_ communities, respectively. Error bars indicate the SEM (*n* = 3).

In the C_3_ community, warming reduced S-DAMO rates (0.195 nmol of ^13^CO_2_ g_dw_^−1^ day^−1^) in comparison to the ambient plot (0.905 nmol of ^13^CO_2_ g_dw_^−1^ day^−1^), while *e*CO_2_ increased rates (4.194 nmol of ^13^CO_2_ g_dw_^−1^ day^−1^) (Fig. 3A, *P* < 0.01). The combination of warming and *e*CO_2_ (+5.1°C + *e*CO_2_) produced rates of S-DAMO higher than both the ambient and the warming-alone treatments (5.33 nmol of ^13^CO_2_ g_dw_^−1^ day^−1^). S-DAMO rates were positively correlated with SO_4_^2−^ concentration (Fig. 3B, *R*^2^ = 0.41, *P* < 0.05). In the C_4_ community, warming also reduced the average S-DAMO activity (0.445 nmol of ^13^CO_2_ g_dw_^−1^ day^−1^) compared to the ambient condition (1.105 nmol of ^13^CO_2_ g_dw_^−1^ day^−1^), but the relationship was weaker (Fig. 3A). Overall, S-DAMO removed 7 and 12% of the CH_4_ produced in the C_3_ and C_4_ communities, respectively (fig. S3B). For the NO_2_^−^- and NO_3_^−^-DAMO pathways, the ^13^CO_2_ production rates from ^13^CH_4_ + NO_2_^−^ and ^13^CH_4_ + NO_3_^−^ treatments were not significantly different from the ^13^CO_2_ production rate of the ^13^CH_4_ treatment alone (fig. S4), indicating that NO_2_^−^ and NO_3_^−^ do not serve as electron acceptors for the AMO process at our study site.

### In situ CH_4_ emission

**Fig. 4.**
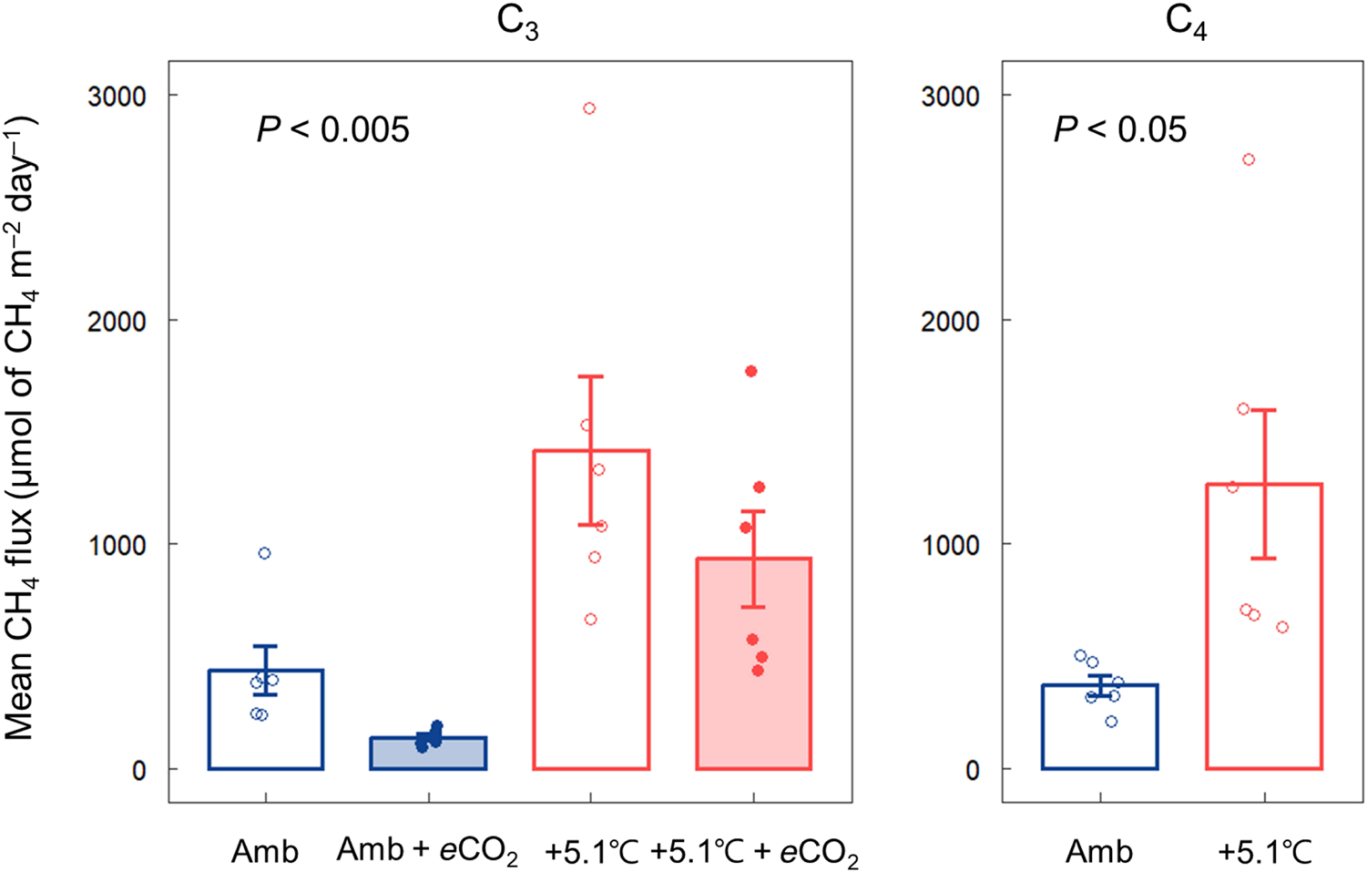
CH_4_ emission in ambient and warmed plots with and without *e*CO_2_. Average CH_4_ emissions measured on two dates in June 2022 in two warming treatments (ambient and ambient + 5.1°C) crossed with *e*CO_2_ (ambient and ambient + 350-ppm CO_2_) in C_3_ and C_4_ communities. A linear mixed-effects model and Mann-Whitney *U* test were used to test the difference in CH_4_ emission between the treatments in the C_3_ and C_4_ communities, respectively. Error bars indicate the SEM (*n* = 6).

In the C_3_ community, warming increased June 2022 CH_4_ emissions, while *e*CO_2_ reduced them (Fig. 4, *P* < 0.005). The combined effect of warming and *e*CO_2_ (+5.1°C + *e*CO_2_) resulted in higher CH_4_ emissions than ambient but lower than in plots with warming alone. CH_4_ emissions from the C_4_ community in June 2022 followed a similar pattern, with warming increasing CH_4_ emissions (Fig. 4, *P* < 0.05). Consistent with the June 2022 trend, the average CH_4_ emissions from 2017 to 2022 showed increased emissions in warmed plots and decreased emissions in *e*CO_2_ plots (fig. S3A, *P* < 0.001).

### Abundance of ANME-1 and ANME-2c

**Fig. 5.**
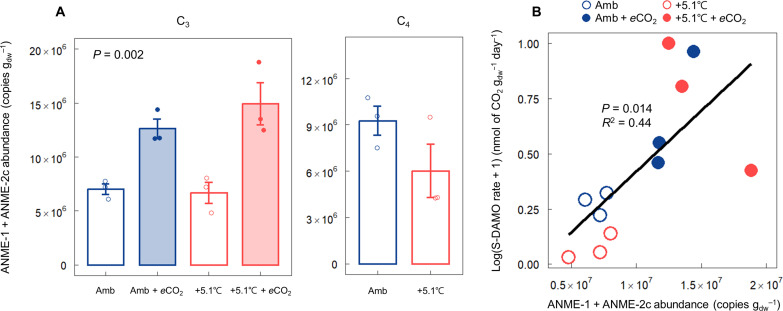
Abundance of S-DAMO–associated microorganisms in ambient and warmed plots with and without *e*CO_2_. (**A**) Combined abundance of ANME-1 and ANME-2c in two warming treatments (ambient and ambient + 5.1°C) crossed with *e*CO_2_ (ambient and ambient + 350-ppm CO_2_) in C_3_ and C_4_ communities. (**B**) Positive correlation between S-DAMO rate and the combined abundance of ANME-1 and ANME-2c in the C_3_ community. A one-way ANOVA and Mann-Whitney *U* test were used to test the difference in combined abundance of ANME-1 and ANME-2c between the treatments in C_3_ and C_4_ communities, respectively. Error bars indicate the SEM (*n* = 3).

In the C_3_ community, *e*CO_2_ increased the sum of ANME-1 and ANME-2c gene abundance, while warming alone had no effect (Fig. 5A, *P* = 0.002). Both ANME-1 and ANME-2c exhibited similar patterns (fig. S5, A and B, *P* < 0.05). A positive correlation was also observed between S-DAMO rate and combined gene abundance of ANME-1 and ANME-2c (Fig. 5B, *R*^2^ = 0.44, *P* = 0.014), with a clear separation of abundance at ambient (low) and *e*CO_2_ (high) (Fig. 5A). The abundances of the two genes considered separately were also positively correlated with S-DAMO rate (fig. S5, C and D, *R*^2^ = 0.28 to 0.48, *P* = 0.01 to 0.1). In the C_4_ community, warming tended to decrease the average abundances of both ANME-1 and ANME-2c (fig. S5, A and B). In addition, the combined gene abundances of ANME-1 and ANME-2c in warming plots were slightly lower compared to those in ambient plots (Fig. 5A).

## DISCUSSION

Variation in SO_4_^2−^ concentrations across plots is not attributable to differences in SO_4_^2−^ inputs, as tidal water is the primary source of SO_4_^2−^ across these colocated plots. Rather, differences in SO_4_^2−^ concentration across treatments are likely due to shifts in rates of SO_4_^2−^ reduction and SO_4_^2−^ regeneration through sulfide (H_2_S) oxidation. Warming decreased SO_4_^2−^ concentration (Fig. 1 and fig. S1) and increased H_2_S concentration (fig. S6) in both the C_3_ and C_4_ communities, suggesting a temperature-driven increase in SO_4_^2−^ reduction rates that was not fully compensated by increased H_2_S oxidation rates. This response is consistent with previous studies showing an exponential increase in SO_4_^2−^ reduction rates with temperature in brackish tidal marshes and intertidal zones (*46*, *47*). This is consistent with a long-term warming experiment in coastal Baltic Sea sediments that related an increase in SO_4_^2−^ reduction to an increase in the relative abundance of SO_4_^2−^-reducing bacteria (*48*). The decrease in redox potential observed in warming plots (Fig. 2A) further suggests that reduction processes were favored over oxidation processes under warming. This trend could be due to a higher temperature sensitivity for reduction than for oxidation, as shown in previous work (*49*), where Fe reduction was more responsive to warming than Fe oxidation. We hypothesize that this pattern extends to other coupled redox processes, such as SO_4_^2−^ reduction and oxidation. This view is supported by greater SO_4_^2−^ depletion in warming plots (fig. S2), suggesting a net shift toward reduction over oxidation under warming compared to ambient temperature. Thus, we propose that differences in temperature sensitivity between SO_4_^2−^ reduction and H_2_S oxidation explain some of the observed porewater chemistry responses to warming.

In contrast to the impact of warming, *e*CO_2_ increased SO_4_^2−^ concentration (Fig. 1 and fig. S1) and decreased H_2_S concentration (fig. S6) in the C_3_ community, a response that coincided with increased soil redox potential and fine-root productivity (Fig. 2 and fig. S7). It is well documented that *e*CO_2_ stimulates root productivity in terrestrial ecosystems (*16*, *50*, *51*) and enhanced root productivity in this experiment induced by *e*CO_2_ is associated with an increase in soil redox potential, presumably due to greater plant-mediated O_2_ transport from the atmosphere into the soil (*16*). We propose that an increase in the O_2_ supply to soils facilitated by the positive C_3_ plant growth response to *e*CO_2_ substantially shifted the net balance between SO_4_^2−^ reduction and regeneration toward regeneration (*52*). This interpretation is consistent with the positive correlation between redox potential and SO_4_^2−^ concentration across treatments (Fig. 2B).

SO_4_^2−^ concentrations in the warming plots were increased by *e*CO_2_ at the 20-cm soil depth but not at deeper depths (Fig. 1). The surficial 0 to 30 cm of the soil profile is also the peak of plant belowground biomass [70 to 95% of root biomass (*53*)] and soil redox potential [−150 to 150 mV (*16*)]. Tidal flooding maintains the soil profile at or near saturation to the soil surface continuously, suggesting that plant gas transport is the dominant source of O_2_ into the soil profile. *e*CO_2_ stimulation of plant O_2_ transport is expected to be dominant at the soil surface layer, leading to an increase in SO_4_^2−^ concentration at shallow depths compared to ambient plots. Because deeper soils are less affected by root activity, they are expected to show relatively small plant O_2_ transport responses to *e*CO_2_, so the warming-induced stimulation of SO_4_^2−^ reduction in deeper soils should favor lower SO_4_^2−^ concentrations. The depth dependence of our treatments on SO_4_^2−^ dynamics is a specific mechanism by which plant-microbe interactions indirectly regulate greenhouse gas emissions. Considering that contemporary climate change leads to both warming and *e*CO_2_ and most biological activity occurs at the soil surface, the influence of plant-microbe interactions on greenhouse gas emissions operating through redox-active elements is important to elucidate in terrestrial ecosystems generally.

As we hypothesized, S-DAMO was the primary AMO process, whereas N-DAMO played a minor role. The decreased production of ^13^CO_2_ in treatments with NO_2_^−^ and NO_3_^−^ addition (fig. S4) suggests that the addition of NO_2_^−^/NO_3_^−^ compounds reduced AMO activity. This finding aligns with previous studies indicating an inhibition of S-DAMO by NO_2_^−^/NO_3_^−^ in marine CH_4_ seeps (*54*) and estuarine sediments (*55*), as denitrification is more thermodynamically and biochemically favorable than S-DAMO. The addition of NO_2_^−^/NO_3_^−^ could potentially have decreased the pH and thereby reduced overall AMO activity; however, no significant changes in pH were observed (fig. S8). Meanwhile, this result is opposed to other studies measuring AMO rates in coastal wetlands, where N-DAMO was identified as the dominant process (*36*, *37*, *39*, *56*). For instance, in the intertidal zones of the East China Sea, N-DAMO accounted for up to 65% of AMO activity, with S-DAMO responsible for the remainder (*36*). Consequently, much of the prior research focused exclusively on N-DAMO, overlooking the crucial role of S-DAMO in coastal wetland ecosystems (*38*, *39*, *56*). These studies were conducted in coastal areas of East China that receive substantial nitrogen inputs from inland sources, including agriculture, industrial emissions, and urban wastewater (*57*, *58*). As a result, these nitrogen inputs provide NO_2_^−^/NO_3_^−^ substrates for N-DAMO activity, which are thermodynamically favored as electron acceptors over SO_4_^2−^ (*32*). However, not all coastal areas are subjected to severe nitrogen inputs from inland (*59*). At our study site, NO_2_^−^ and NO_3_^−^ concentrations in June 2022 were at or below detection limits (fig. S9), likely because of minimal inputs of NO_2_^−^/NO_3_^−^ from the adjacent estuary and soils that are continuously saturated to the soil surface (*60*, *61*).

The increased O_2_ availability triggered by *e*CO_2_ has the potential to suppress AMO activity through toxic effects or competition with aerobic methanotrophs for CH_4_ (*62*), so our initial hypothesis was that *e*CO_2_ would reduce overall AMO activity. Contrary to expectations, *e*CO_2_ instead promoted AMO activity, particularly S-DAMO (Fig. 3A). This suggests that aerobic and anaerobic CH_4_ oxidizers are not competing for CH_4_, which is likely given the high porewater CH_4_ concentrations [~50 to 150 μM (*15*)] at our study site. Rather, a positive correlation between the S-DAMO rate and the redox potential indicates (fig. S10) that O_2_ enhances S-DAMO activity by increasing the SO_4_^2−^ concentration and that both aerobic and anaerobic pathways contribute to mitigating CH_4_ emissions from tidal wetlands. We did not estimate the contribution of AMO to overall CH_4_ oxidation, but S-DAMO contributed up to 5.6% of the total CH_4_ oxidation in a coastal wetland of the East China Sea (*29*).

The stimulation of S-DAMO rates in *e*CO_2_ plots was likely induced primarily by increased rates of H_2_S oxidation to SO_4_^2−^, regenerating the electron acceptor required for S-DAMO respiration. This increase in S-DAMO activity is evidence that SO_4_^2−^ availability limits S-DAMO rates, which is consistent with the relatively low salinity (~8) and associated SO_4_ availability at our site. This is supported by the positive correlation between the S-DAMO rate and the SO_4_^2−^ concentration (Fig. 3B) in the C_3_ community. Furthermore, SO_4_^2−^ concentration has been shown to be positively correlated with the OTU number, the Shannon index, and the Chao1 index of ANME archaea in coastal wetlands, further indicating that SO_4_^2−^ availability generally limits S-DAMO activity in coastal wetlands (*36*). While earlier studies have suggested that S-DAMO microbes might also use alternative substrates such as manganese, iron, and organic humic substances when SO_4_^2−^ is scarce, these alternatives appear to lead to lower rates of AMO. In the present case, only humic substances could have served as alternative electron acceptors in these highly organic (~80% organic matter) soils.

We observed an increase in the gene abundance of ANME-1 and ANME-2c, microorganisms involved in S-DAMO, under *e*CO_2_ conditions (Fig. 5A and fig. S5). ANME-1 showed a higher abundance and a stronger correlation with S-DAMO rates than ANME-2c. This may reflect ecological distinctions between the two groups. ANME-2c forms stable aggregates with SO_4_^2−^-reducing bacteria, facilitating CH_4_ oxidation (*63*, *64*). We hypothesize that *e*CO_2_-induced plant O_2_ transport suppressed SO_4_^2−^-reducing bacteria, which thrive under anaerobic conditions (*65*), thereby reducing ANME-2c’s S-DAMO capacity. In contrast, ANME-1 uses an independent S-DAMO strategy that does not rely on SO_4_^2−^-reducing bacteria (*66*), making it less affected by O_2_ intrusion and giving it a competitive advantage.

As we hypothesized, +5.1°C warming decreased S-DAMO activity compared to ambient conditions (Fig. 3A), likely a consequence of decreased SO_4_^2−^ availability. However, when warming was combined with *e*CO_2_ in the C_3_ community, S-DAMO activity rebounded to rates similar to those in the ambient temperature treatments, presumably due to increased rates of plant-mediated O_2_ transport supporting SO_4_^2−^ regeneration. This interpretation is supported by the significant positive correlation between SO_4_^2−^ concentration and S-DAMO rate (Fig. 3B). However, the magnitude of the S-DAMO response to our treatments, which are on a log scale, was much larger than the SO_4_^2−^ concentration responses (compare Figs. 1A and 3A), and the correlation (Fig. 3B, *R*^2^ = 0.41) indicated substantial unexplained variation. Because SO_4_^2−^ concentration is the balance of two opposing and rapid processes—SO_4_^2−^ reduction and H_2_S oxidation—it only approximates SO_4_^2−^ availability. A more precise covariate for S-DAMO would be the direct measurement of SO_4_^2−^ reduction and H_2_S oxidation rates.

The response of CH_4_ emissions to climate change treatments follows the observed S-DAMO rate responses, suggesting that reduced S-DAMO under warming contributed to higher CH_4_ emissions and increased S-DAMO under *e*CO_2_ contributed to lower CH_4_ emissions (Fig. 4 and fig. S3A). These results suggest that S-DAMO plays a crucial role in regulating CH_4_ emissions in coastal wetlands. In the relatively low salinity (~8) setting of our study site, we estimated that S-DAMO removes 7 and 12% of CH_4_ produced by methanogenesis in the C_3_ and C_4_ communities, respectively (fig. S3B), a meaningful fraction given the large sustained global warming potential of CH_4_ (*67*). However, given estimates from other sites that S-DAMO can consume up to 90% of CH_4_ production in some tidal ecosystems (*22*), the potential for even larger climate change effects in the greenhouse gas balance of coastal wetlands is substantial.

**Fig. 6.**
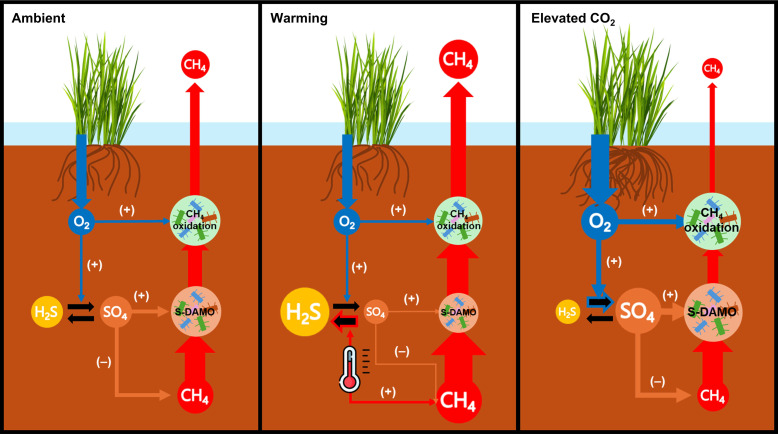
Conceptual model of the mechanisms that regulate CH_4_ emissions in response to climate change in a coastal wetland. Warming and *e*CO_2_ influence CH_4_ emissions through changes in O_2_ transport and SO_4_^2−^ dynamics. The size of the arrows indicates the magnitude of the rate, and the size of the circles indicates the size of the pool. Thermometer icon indicates the effect of the warming temperature treatment.

Most previous studies on SO_4_^2−^ regulation of ecosystem CH_4_ emissions focus on competitive interactions between SO_4_^2−^-reducing bacteria and methanogens. Our study offers the perspective that SO_4_^2−^ availability can also modulate ecosystem CH_4_ emissions by regulating S-DAMO activity (Fig. 6). We previously reported that warming substantially increased CH_4_ emissions by stimulating methanogenic activity, while *e*CO_2_ reduced it by promoting O_2_ transport belowground where it supports aerobic methanotrophs (*16*). Our findings add an anaerobic oxidation mechanism to explain these observations, which is that warming increases CH_4_ emissions by reducing S-DAMO activity, while *e*CO_2_ decreases CH_4_ emissions by stimulating S-DAMO activity. This mechanism is consistent with our previous hypothesis (*16*) that *e*CO_2_ enhances CH_4_ oxidation through increased plant-mediated O_2_ transport but through more complex mechanisms that link carbon cycling to sulfur cycling. The relative contributions of aerobic and anaerobic methanotrophy to this important CH_4_ sink remain to be determined, along with related questions about the fate of O_2_ in the plant rhizosphere where there are multiple competing chemical and biological sinks for O_2_. While the present study linked plant trait effects on CH_4_ oxidation to sulfur oxidation, the mechanism for these plant-microbe interactions is transferable to other wetland ecosystems where the stimulation of plant-mediated O_2_ transport by *e*CO_2_ or other means would instead increase the availability of oxidants such as NO_3_^−^, Fe(III), or oxidized humic substances (*49*, *68*). Understanding these dynamics and integrating them into predictive models are essential for forecasting the future impact of coastal wetlands on global CH_4_ budgets and formulating strategies to mitigate CH_4_ emissions in the context of climate change.

## MATERIALS AND METHODS

### Site description and experimental design

The study was conducted at the Smithsonian’s GCReW, a brackish high marsh located on the western shore of the Chesapeake Bay, USA (38°55′N, 76°33′W), using the SMARTX (*45*). This comprehensive experiment includes 30 plots distributed over six warming transects; each plot is 2 m by 2 m and is encircled by a 0.2-m buffer zone to minimize edge effects. Half of the transects are located in a high-elevation area dominated by *Spartina patens* and *Distichlis spicata* (referred to as the C_4_ community), which experiences flooding during 10 to 20% of high tides. The remaining transects are located in a low-elevation area dominated by *Schoenoplectus americanus* (referred to as the C_3_ community) with flooding occurring during 30 to 60% of high tides. Soils are highly organic (<80% organic matter) to a depth of 5 m, which is a characteristic common in high marshes of the Chesapeake Bay and other regions (*15*). The low mineral content (<20%) influences CH_4_ dynamics due to the negligible competition between methanogens and iron-reducing bacteria for electron donors, attributed to the lack of a substantial amount of poorly crystalline iron oxides (*69*), as has been previously observed at this site (*70*). Species that dominate the two communities represent two fundamental photosynthetic pathways that respond either weakly (C_4_) or strongly (C_3_) to *e*CO_2_. For that reason, the C_3_ community has an additional *e*CO_2_ treatment.

In this study, a subset of 18 plots was used, including 12 from the C_3_ community and 6 from the C_4_ community. The C_3_ community uses a complete factorial design, featuring two temperature levels (ambient and +5.1°C warming) and two CO_2_ levels (ambient and +350 ppm). The C_4_ community consists of two temperature levels (ambient and +5.1°C). Each combination of treatment conditions is replicated three times.

Warming in the plots is achieved using infrared heaters to increase aboveground plant-surface temperature and vertical resistance cables to raise soil temperature to a depth of 1.5 m (*15*, *16*). Temperature control is maintained through an integrated microprocessor-based feedback system (*16*, *71*). In addition, the *e*CO_2_ plots are equipped with 2-m-diameter open-top chambers, allowing for independent CO_2_ concentration control within each chamber. The warming began in June 2016 and operates continuously throughout the year, while the *e*CO_2_ treatment started in April 2017, applied only during daylight hours in the growing season (April to November).

### Porewater chemistry and fine-root productivity

Porewater samples were collected in May and July 2022 through stainless-steel “sippers” installed permanently in each plot in 2016 (*15*). In each plot, duplicate clusters of sippers were installed at depths of 20, 40, 80, and 120 cm below the soil surface. On collection days, the stagnant porewater within the sippers was initially expelled, followed by the extraction of 60 ml of porewater from each specified depth (30 ml from each sipper) and stored in syringes fitted with three-way stopcocks. From each sample, a 10-ml aliquot was passed through a prerinsed 0.45-μm syringe-mounted filter, preserved with 5% zinc acetate and sodium hydroxide, and frozen for future SO_4_^2−^ and chloride (Cl^−^) analysis.

The concentrations of SO_4_^2−^ and Cl^−^ were analyzed using a Dionex Integrion system, equipped with an A11 4-μm fast column operated with a 35 mM KOH eluent. The degree of SO_4_^2−^ depletion was computed from the porewater concentrations of SO_4_^2−^ (SO4_pw_) and Cl^−^ (Cl_pw_), using the constant molar ratio of Cl^−^ to SO_4_^2−^ in surface seawater at the study site (*R*_sw_ = 6.84) as per the equation: SO_4_^2−^ depletion = Cl_pw_/*R*_sw_ – SO4_pw_ (*72*). Normally, if influenced solely by seawater contribution, then the Cl to SO_4_^2−^ ratio stays unchanged. However, in coastal wetland ecosystems, SO_4_^2−^ reduction by SO_4_^2−^-reducing bacteria and regeneration of SO_4_^2−^ by H_2_S oxidation may modify this balance, making SO_4_^2−^ depletion a useful indicator of the in situ rates of SO_4_^2−^ dynamics.

The redox potential in the C_3_ community was measured using an automated redox system (*16*). Briefly, six redox probes with platinum bands were installed at a depth of 20 cm in each plot. Measurements were taken every 30 min, and data from May to July 2022 were used in this study. Replicate measurements within a plot were averaged for each time point (*n* = 6) and then averaged again across plots (*n* = 48) to obtain a daily mean per plot. For statistical analysis, daily means were averaged per plot from May to June 2022.

Fine-root productivity was measured using yearlong root ingrowth cores (0 to 40 cm in depth), following the method described previously (*45*), with three replicate cores per plot. Briefly, roots and rhizomes were collected, sorted by functional groups, oven-dried at 60°C, and weighed.

### Potential AMO, CH_4_ production, and in situ CH_4_ emission

Potential AMO activity was evaluated using the ^13^C stable isotope technique (*28*). Soil samples were collected from each plot in June 2022, at depths between 15 and 30 cm. On the day of collection, slurries were prepared by mixing 15 g of soil from each plot with 30 ml of porewater, sourced from near the SMARTX at a depth of 20 cm, and these were then placed into 120-ml vials. Before its use, the porewater was passed through a 0.2-μm membrane filter and degassed with N_2_. These vials were purged with N_2_ and were subjected to a preincubation for 5 days in a shaker at 150 rpm and 20°C in darkness, a process designed to acclimate the microbial communities to the ambient temperature and to eliminate any residual O_2_ and NO*_x_*^−^.

Following the preincubation, the vials were again purged with N_2_ and organized into five distinct experimental groups: (i) ^12^CH_4_, (ii) ^13^CH_4_ (99.9% ^13^C; Sigma-Aldrich, DE), (iii) ^13^CH_4_ + SO_4_^2−^ (final SO_4_^2−^ concentration of initial porewater + 30 mM), (iv) ^13^CH_4_ + NO_2_^−^ (final NO_2_^−^ concentration of 0.5 mM), and (v) ^13^CH_4_ + NO_3_^−^ (final NO_3_^−^ concentration of 5 mM). In each vial, 20 μl of Na_2_SO_4_, NaNO_2_, or NaNO_3_ was added, as applicable. Moreover, 80 μl of CH_4_ [^12^CH_4_ for group i and ^13^CH_4_ for groups ii to v] were infused to reach a target headspace CH_4_ concentration of 1% (v/v). Each treatment was performed in triplicate. Additional vials were set up to monitor the headspace O_2_ concentration. Five days after the start of incubation, the headspace from each vial was collected and transferred to 12-ml evacuated double-wadded exetainers (Labco, UK) for analysis. Except for the N_2_ purging, all procedures were conducted in an anaerobic chamber with an atmosphere of 97% N_2_ and 3% H_2_. The total CO_2_ concentration was measured using gas chromatography (Varian, USA), and the δ^13^C ratio of CO_2_ was measured using an isotope ratio mass spectrometer (Thermo Fisher Scientific, Germany) at the University of California Davis Stable Isotope Facility. In addition, the headspace CH_4_ concentration of control treatments was measured using gas chromatography (Varian, USA) to determine the potential CH_4_ production rate. Using the rate, we calculated the proportion of S-DAMO to gross CH_4_ production.

The potential activities of S-DAMO, NO_2_^−^-DAMO, and NO_3_^−^-DAMO were quantified on the basis of ^13^CO_2_ production during incubation. Specifically, the S-DAMO rate was determined by the difference in ^13^CO_2_ production between ^13^CH_4_-only and ^13^CH_4_ + SO_4_^2−^ treatments. NO_2_^−^-DAMO and NO_3_^−^-DAMO rates were determined by the ^13^CO_2_ production difference between ^13^CH_4_ and ^13^CH_4_ + NO_2_^−^ and ^13^CH_4_ + NO_2_^−^ and ^13^CH_4_ + NO_3_^−^ treatments, respectively.

In situ CH_4_ emissions were measured as described previously (*15*). Briefly, CH_4_ fluxes were measured from March to November from 2017 to 2022 using static chambers, and fluxes were calculated as the linear rate of change in CH_4_ concentration within the chamber headspace over 5 min.

### DNA extraction and quantitative polymerase chain reaction

Microbial DNA and RNA were coextracted from 2 g of soil samples using the RNeasy PowerSoil Total RNA Kit and the RNeasy PowerSoil DNA Elution Kit according to the manufacturer’s instructions (QIAGEN, Hilden, Germany), and only the extracted DNA was used in this study. The final DNA pellet was delicately suspended in 30 μl of deoxyribonuclease-free water. Last, the quantity and purity of DNA were assessed using the Qubit 4.0 fluorometer and NanoDrop ND-1000 spectrometer (Thermo Fisher Scientific, Waltham, USA).

The abundances of ANME archaea were estimated using quantitative polymerase chain reaction (qPCR) with SYBR Green Real-Time PCR Master Mix (Toyobo, Osaka, Japan) and CFX Opus 96 Real-Time PCR System (Bio-Rad, CA, USA), targeting small subunit ribosomal RNA genes for ANME-1 and ANME-2c. Primers specific to each gene are detailed in table S1. Each reaction mixture, with a total volume of 10 μl, was composed of 3.6 μl of distilled water, 5 μl of SYBR Green Master Mix, 0.2 μl of each forward and reverse primer (0.2 μM), and 1 μl of DNA solution. The copy number of genes was calculated using standard curves derived from serial 10-fold dilutions of gene fragments. For all qPCR reactions, the standard curves exhibited an efficiency greater than 98% and a *R*^2^ higher than 0.99. Amplification specificity for each qPCR reaction was confirmed through the analysis of melting curves and 1.3% agarose gel electrophoresis.

### Statistical analysis

All statistical analyses were performed using R (version 4.3.3). Linear mixed-effects models were used to evaluate the relationships between the S-DAMO rate and the abundance of ANME-1, the abundance of ANME-2, the combined abundance of ANME-1 and ANME-2c, CH_4_ emission, and between redox potential, soil pH, H_2_S concentration, and SO_4_^2−^ concentration in the C_3_ community. Climate factors (ambient, +5.1°C, *e*CO_2_, and +5.1°C + *e*CO_2_) were incorporated as random effects to account for between-treatment variability. An exponential regression analysis was used to test the relationship between the S-DAMO rate and the SO_4_^2−^ concentration, as well as between the S-DAMO rate and the SO_4_^2−^ depletion. Linear mixed-effects models were used to assess the effect of warming and *e*CO_2_ on SO_4_^2−^ concentration, SO_4_^2−^ depletion, and redox potential in the C_3_ community. Climate factors were used as categorical variables, and month was used as random effects. One-way analysis of variance (ANOVA) was used to test the difference in S-DAMO rate, the abundance of ANME-1, ANME-2c, fine-root productivity, and combined abundance of ANME-1 and ANME-2c between the treatments in the C_3_ community. Mann-Whitney *U* tests were used to test the difference in S-DAMO rate, abundance of ANME-1 and ANME-2c, combined abundance of ANME-1 and ANME-2c, soil pH, H_2_S concentration, SO_4_^2−^ concentration, SO_4_^2−^ depletion, fine-root productivity, and CH_4_ emission between the treatments in the C_4_ community. We did not include post hoc labels in the figure to indicate significant differences between treatments, as the large variability and small sample size reduced statistical power, making it difficult to detect true differences (*73*).

## Supplementary Materials

### This PDF file includes:

Figs. S1 to S10 Table S1 References
