# Supplementary material for: Climate-induced shifts in sulfate dynamics regulate anaerobic methane oxidation in a coastal wetland
Source: Sci Adv. 2025 Apr 23;11(17):eads6093. doi: 10.1126/sciadv.ads6093 (PMC12017331; doi:10.1126/sciadv.ads6093)
Supplement: Supplementary file 1 — Figs. S1 to S10 Table S1 References [file sciadv.ads6093_sm.pdf]

Science Advances
AAAS

Supplementary Materials for

Climate-induced shifts in sulfate dynamics regulate anaerobic methane oxidation in a coastal wetland

Jaehyun Lee *et al.*

Corresponding author: Jaehyun Lee, jaehyunlee@kist.re.kr; J. Patrick Megonigal, megonigalp@si.edu

Sci. Adv. **11**, eads6093 (2025)
DOI: 10.1126/sciadv.ads6093

This PDF file includes:

Figs. S1 to S10
Table S1
References

Fig. S1.

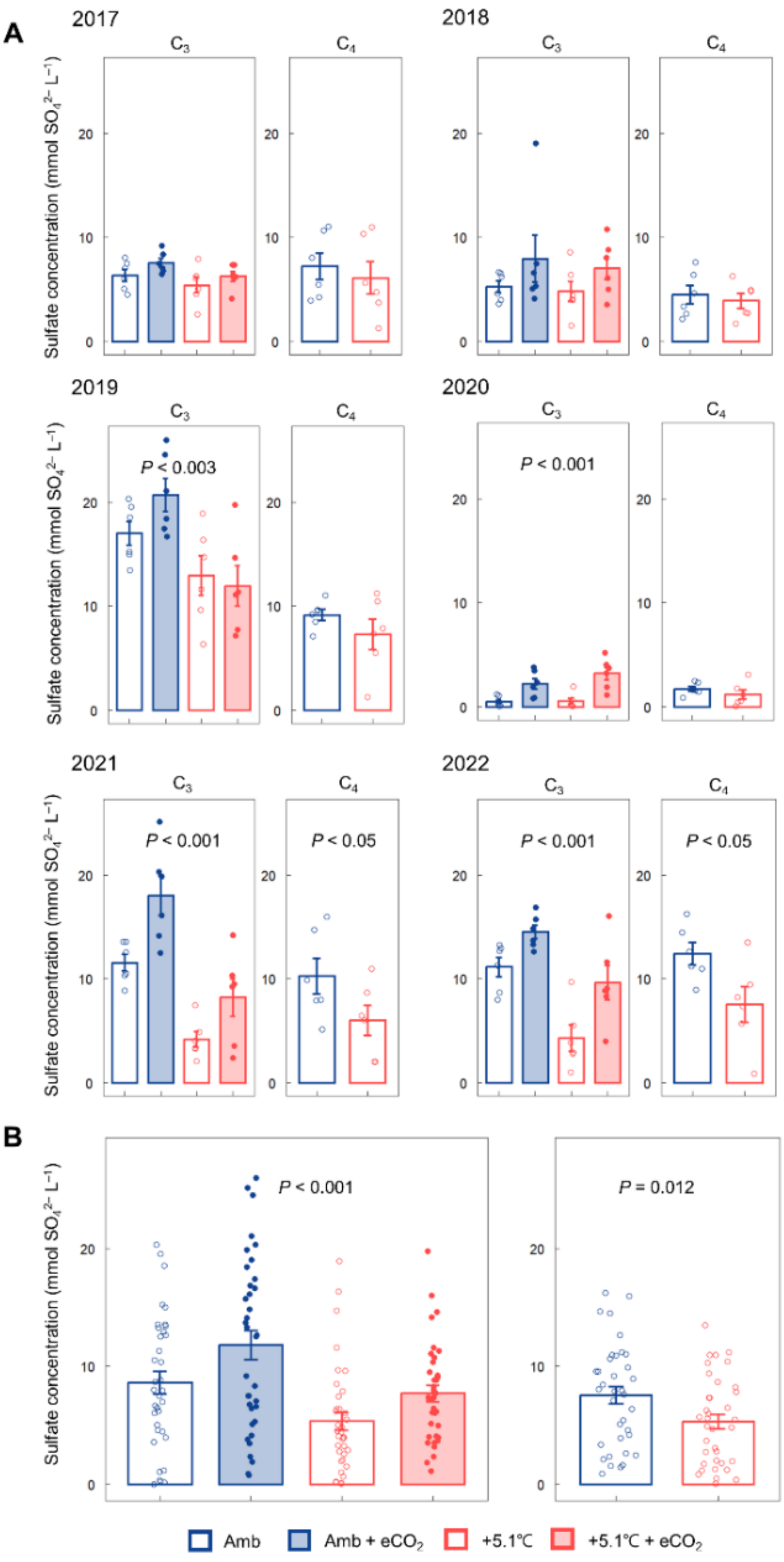
A
2017
2018
2019
2020
2021
2022
C3
C4
Sulfate concentration (mmol SO4 2− L−1)
P < 0.003
P < 0.001
P < 0.05
B
P = 0.012
Amb
Amb + eCO2
+5.1℃
+5.1℃ + eCO2

Fig. S1. Porewater sulfate concentration in ambient and warmed plots with and without elevated CO_2 across the years. (**A**) Porewater SO_4^{2-} concentration measured in May and July from 2017 to 2022 at depth of 20 cm in t two warming treatments (ambient, ambient + 5.1 °C) crossed with elevated CO_2 (ambient, ambient + 350 ppm CO_2). (**B**) Average porewater SO_4^{2-} concentration in May and July from 2017 and 2022 at depth of 20 cm. A linear mixed effect model and Mann-Whitney U test were used to test the difference in SO_4^{2-} concentration between the treatments in C_3 and C_4 communities, respectively. Error bars indicate the standard error of the mean ($N = 6$ for panel (**A**) and $N = 36$ for panel (**B**)).

Fig. S2.

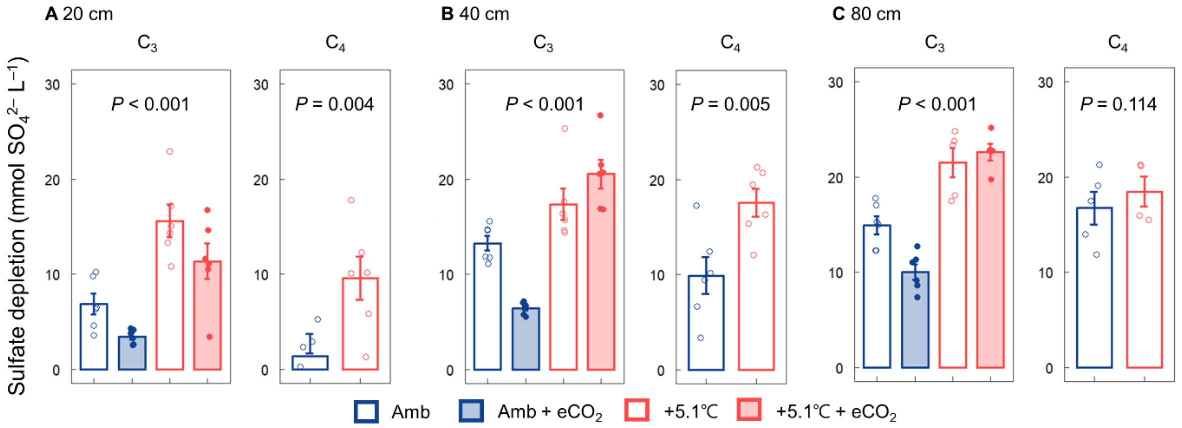

Fig. S2. Porewater sulfate depletion in ambient and warmed plots with and without elevated CO_2 across the different depths. Porewater SO_4^{2-} depletion measured in May and July of 2022 at depth of (**A**) 20 cm, (**B**) 40 cm, and (**C**) 80 cm in two warming treatments (ambient, ambient + 5.1 °C) crossed with elevated CO_2 (ambient, ambient + 350 ppm CO_2) in C_3 and C_4 communities. A linear mixed effect model and Mann-Whitney U test were used to test the difference in SO_4^{2-} depletion between the treatments in C_3 and C_4 communities, respectively. Error bars indicate the standard error of the mean (N = 6).

Fig. S3.

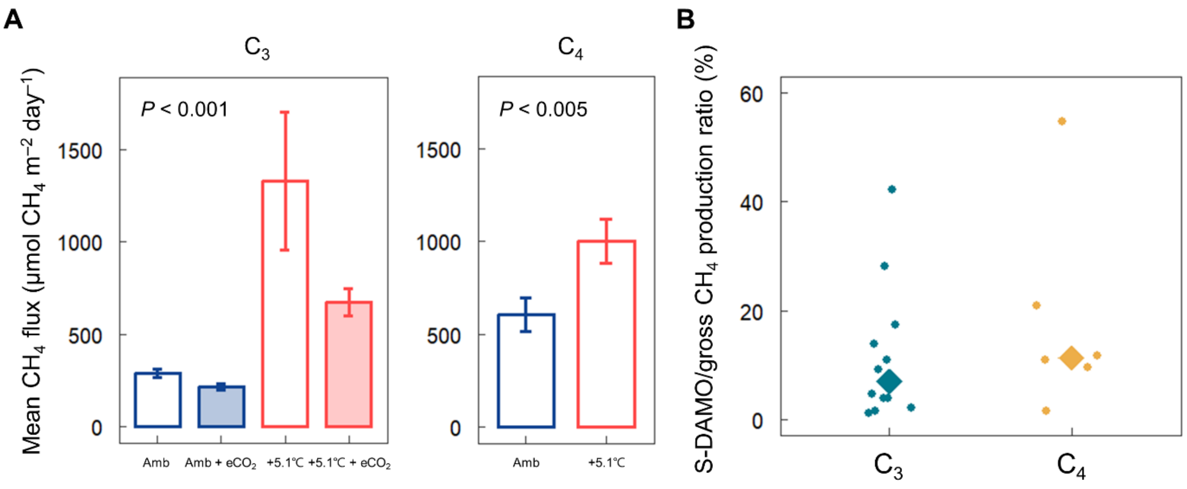

Fig. S3. Average CH_4 emissions and the proportion of CH_4 removal by sulfate-dependent anaerobic CH_4 oxidation. (**A**) Average CH_4 emission from 2017 to 2022 in two warming treatments (ambient, ambient + 5.1 °C) crossed with elevated CO_2 (ambient, ambient + 350 ppm CO_2) in C_3 and C_4 communities. (**B**) Contribution of sulfate-dependent anaerobic CH_4 oxidation (S-DAMO) to gross CH_4 production. Error bars indicate the standard error of mean (N = 75) and large diamonds denote the median values.

Fig. S4.

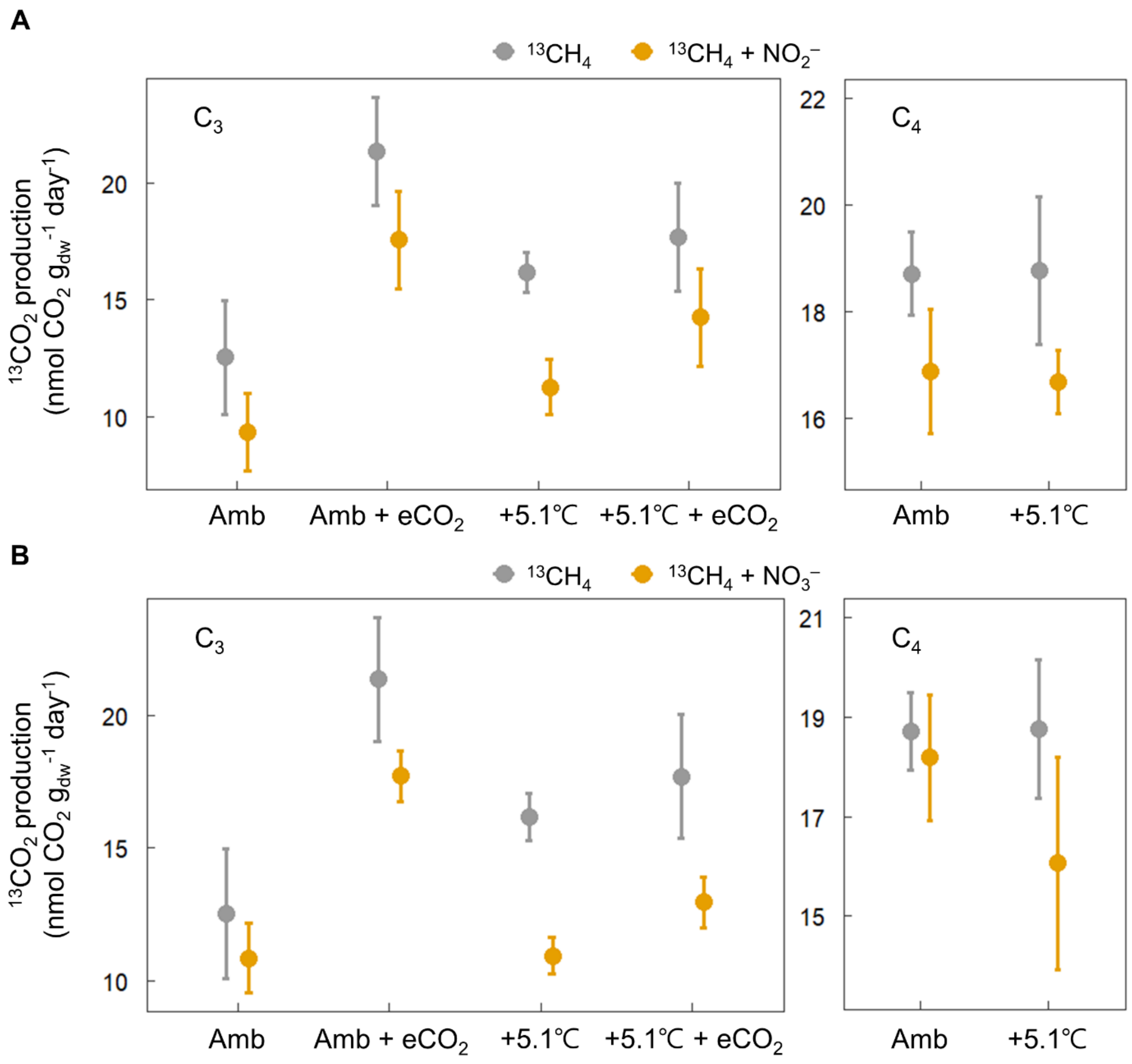

Fig. S4. $^{13}CO_2$ production rate for N-DAMO process. $^{13}CO_2$ production rate of (**A**) $NO_2^–$ amended and (**B**) $NO_3^–$ amended in two warming treatments (ambient, ambient + 5.1 °C) crossed with elevated CO_2 (ambient, ambient + 350 ppm CO_2) in C_3 and C_4 communities. Error bars indicate the standard error of the mean (N = 3).

Fig. S5.

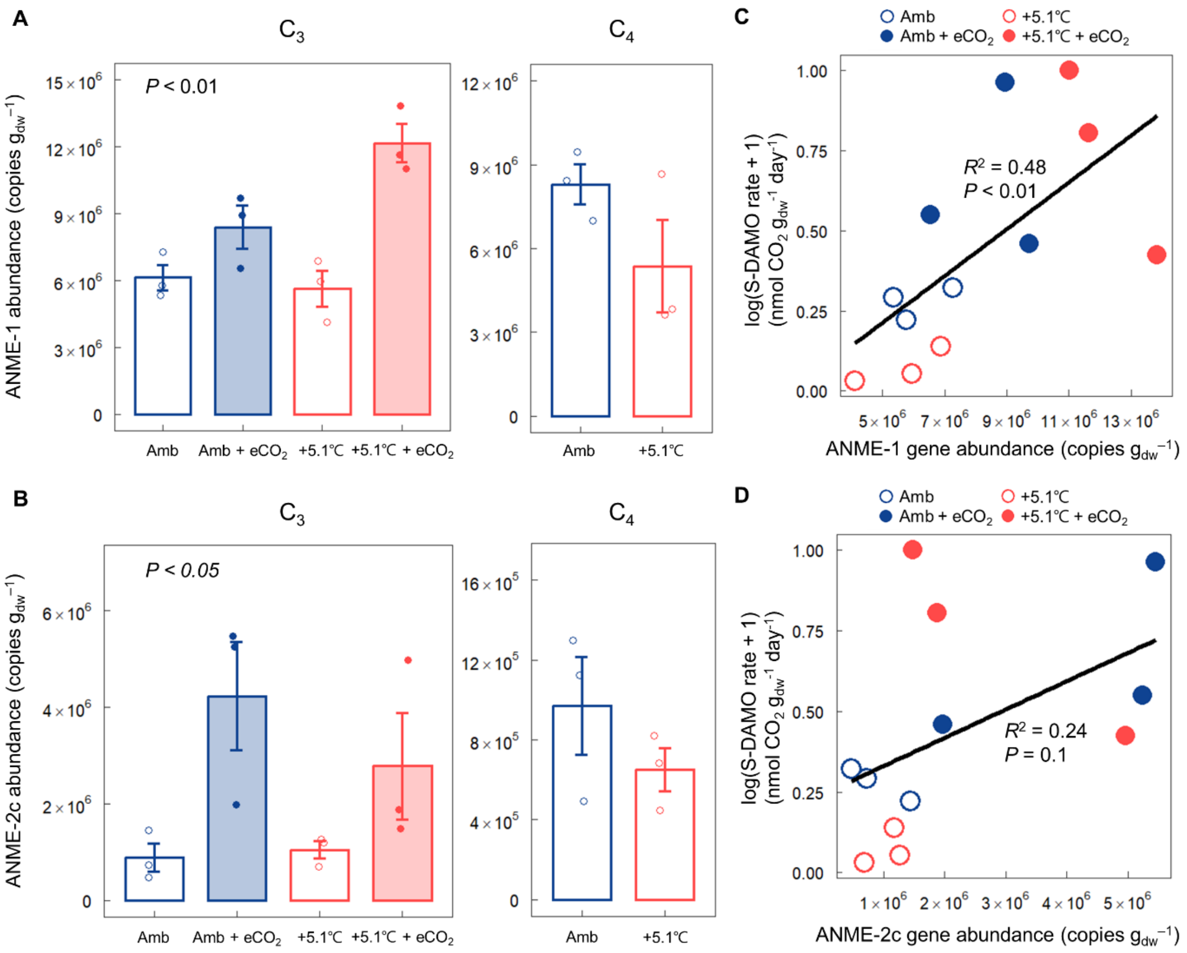

Fig. S5. Gene abundance of S-DAMO associated microorganisms in ambient and warmed plots with and without elevated CO_2. Gene abundance of (**A**) ANME-1 and (**B**) ANME-2c in two warming treatments (ambient, ambient + 5.1 °C) crossed with elevated CO_2 (ambient, ambient + 350 ppm CO_2) in C_3 and C_4 communities. (**C**) Positive relationship between S-DAMO rate and the gene abundance of ANME-1, and (**D**) between S-DAMO rate and the gene abundance of ANME-2c. A one-way ANOVA and Mann-Whitney U test were used to test the difference in ANME-1 and ANME-2c abundances between the treatments in C_3 and C_4 communities, respectively. Error bars indicate the standard error of the mean (N = 3).

Fig. S6.

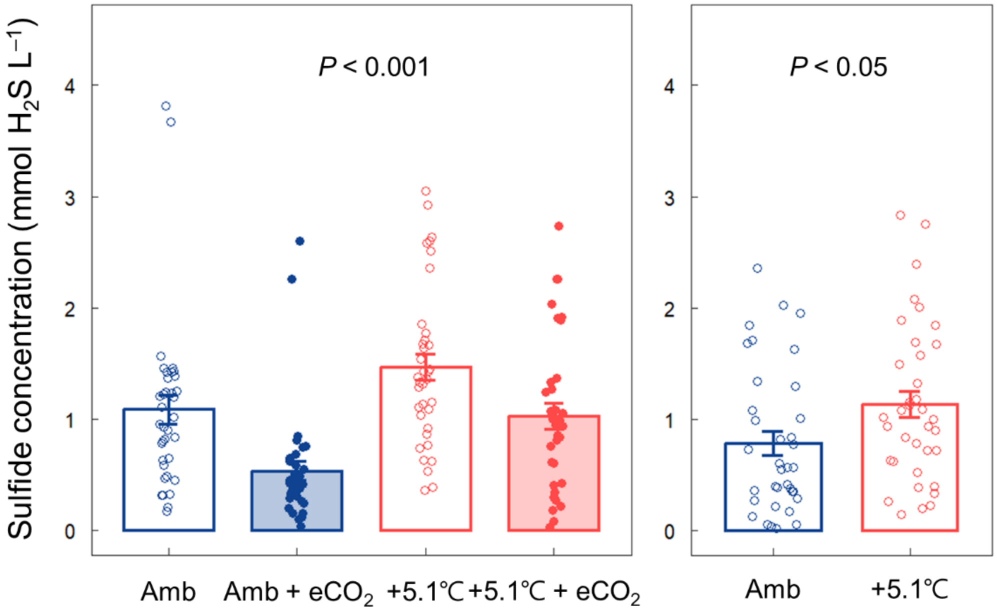

Fig. S6. Sulfide concentration in ambient and warmed plots with and without elevated CO_2. Average H_2S concentration measured at a depth of 20 cm in May and July from 2017 to 2022 in two warming treatments (ambient, ambient + 5.1 °C) crossed with elevated CO_2 (ambient, ambient + 350 ppm CO_2) in C_3 and C_4 communities. A linear mixed effect model and Mann-Whitney U test were used to test the difference in sulfide concentration between the treatments in C_3 and C_4 communities, respectively. Error bars indicate the standard error of the mean (N = 36).

Fig. S7.

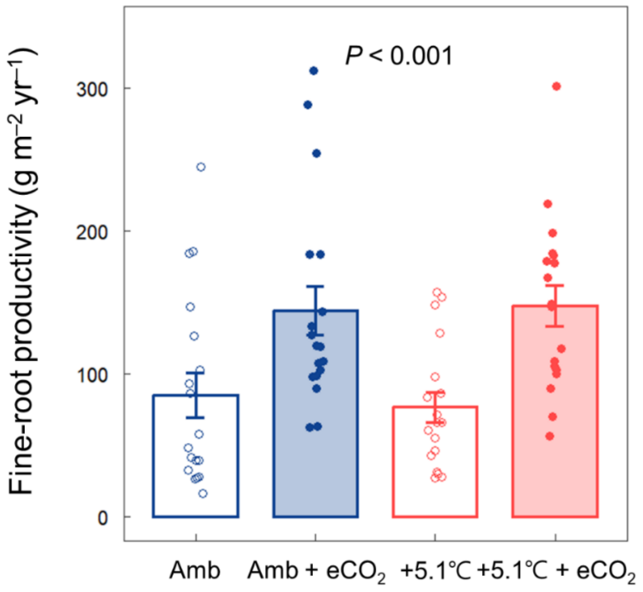

Fig. S7. Fine-root productivity in ambient and warmed plots with and without elevated CO_2. Average annual fine-root productivity measured from 2017 to 2022 in two warming treatments (ambient, ambient + 5.1 °C) crossed with elevated CO_2 (ambient, ambient + 350 ppm CO_2) in C_3 and C_4 communities. A linear mixed effect model and Mann-Whitney U test were used to test the difference in fine-root productivity between the treatments in C_3 and C_4 communities, respectively. Error bars indicate the standard error of the mean (N = 18).

Fig. S8.

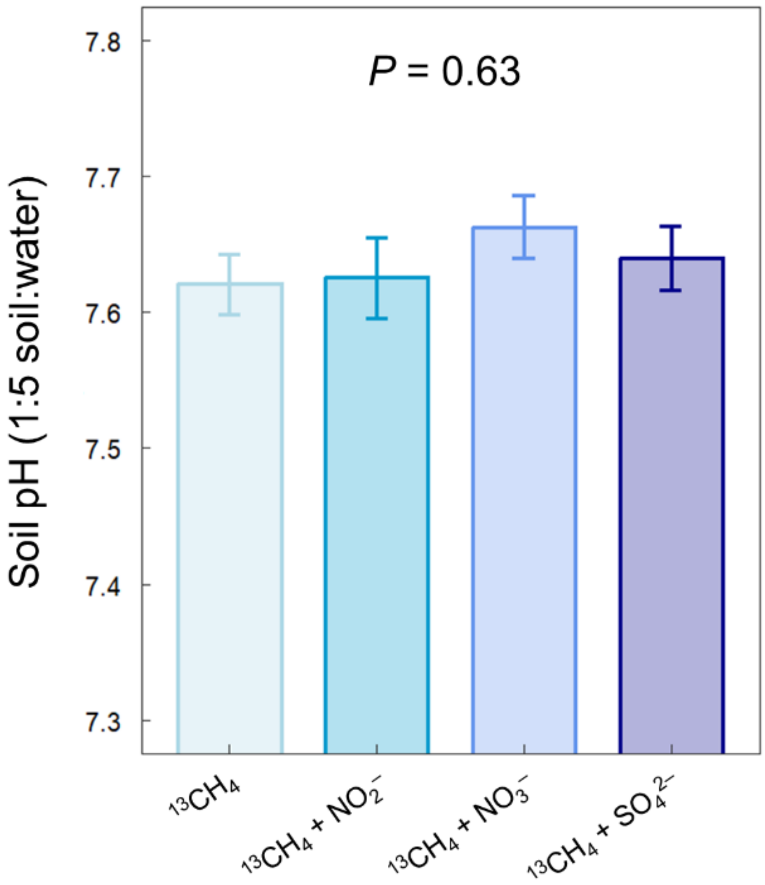

Fig. S8. Soil pH at the end of the incubation experiment. Soil pH following incubation across different electron acceptor additions. A one-way ANOVA was used to test differences in soil pH between the different electron acceptor treatments. Error bars represent the standard error of the mean (N = 18).

Fig. S9.

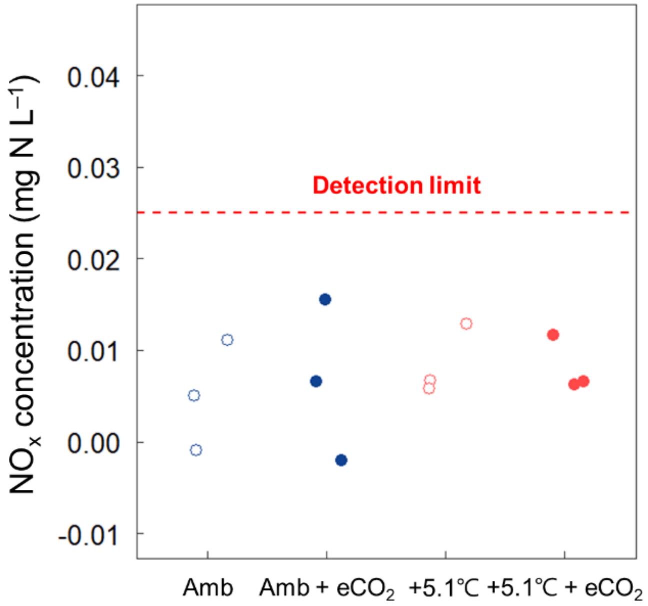

Fig. S9. NO_x concentration in ambient and warmed plots with and without elevated CO_2. NO_x (NO_2^- + NO_3^-) concentration measured in July 2022 at a depth of 20 cm in two warming treatments (ambient, ambient + 5.1 °C) crossed with elevated CO_2 (ambient, ambient + 350 ppm CO_2) in the C_3 community. Red dashed line indicates the detection limit (> 0.025 mg N L^{-1}).

Fig. S10.

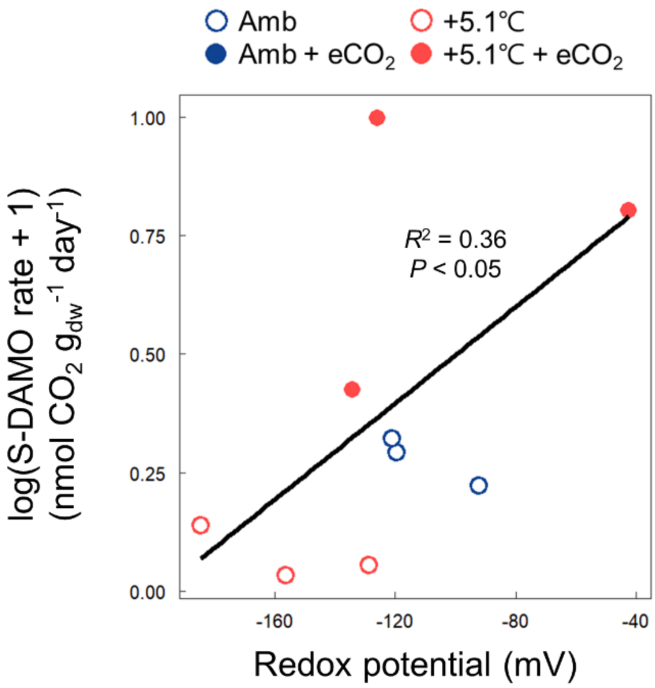

Fig. S10. Relationship between S-DAMO rate and redox potential. Positive correlation between S-DAMO rate and redox potential measured in May to July of 2022 in the C_3 community.

Table S1.

Table S1. Primer sequences for qPCR. Sequences of primer sets used to assess the abundance of ANME-1 and ANME-2c.

Target gene	Primer	Sequence	Reference
ANME-1	ANME-1F	GCT TTC AGG GAA TAC TGC	Lloyd et al. (*74*)
	ANME-1R	TCG CAG TAA TGC CAA CAC	
ANME-2c	ANME-2cF	TCG TTT ACG GCT GGG ACT AC	Vigneron et al. (*75*)
	ANME-2cR	TCC TCT GGG AAA TCT GGT TG	

REFERENCES AND NOTES

1. S. D. Bridgham, J. P. Megonigal, J. K. Keller, N. B. Bliss, C. Trettin, The carbon balance of North American wetlands. *Wetlands* **26**, 889–916 (2006).

2. R. Paudel, N. M. Mahowald, P. G. M. Hess, J. D. Watts, J. S. Kimball, B. Poulter, P. Bousquet, J. G. Canadell, P. Ciais, A. Peregon, V. K. Arora, D. J. Beerling, V. Brovkin, C. D. Jones, F. Joos, C. Peng, S. Peng, C. Prigent, R. Schroeder, W. J. Riley, M. Saito, Global wetland contribution to 2000–2012 atmospheric methane growth rate dynamics. *Environ. Res. Lett.* **12**, 094013 (2017).

3. C. P. Bueno de Mesquita, W. H. Hartman, M. Ardón, S. G. Tringe, Disentangling the effects of sulfate and other seawater ions on microbial communities and greenhouse gas emissions in a coastal forested wetland. *ISME Commun.* **4**, ycae040 (2024).

4. C. P. Bueno de Mesquita, W. H. Hartman, M. Ardón, E. S. Bernhardt, S. C. Neubauer, N. B. Weston, S. G. Tringe, Microbial ecology and site characteristics underlie differences in salinity-methane relationships in coastal wetlands. *J. Geophys. Res. Biogeosci.* **129**, e2024JG008133 (2024).

5. J. A. Rosentreter, D. T. Maher, D. V. Erler, R. H. Murray, B. D. Eyre, Methane emissions partially offset "blue carbon" burial in mangroves. *Sci. Adv.* **4**, eaao4985 (2018).

6. M. P. J. Oreska, K. J. McGlathery, L. R. Aoki, A. C. Berger, P. Berg, L. Mullins, The greenhouse gas offset potential from seagrass restoration. *Sci. Rep.* **10**, 7325 (2020).

7. M. L. Kirwan, J. P. Megonigal, G. L. Noyce, A. J. Smith, Geomorphic and ecological constraints on the coastal carbon sink. *Nat. Rev. Earth Environ.* **4**, 393–406 (2023).

8. J. R. Holmquist, L. Windham-Myers, B. Bernal, K. B. Byrd, S. Crooks, M. E. Gonneea, N. Herold, S. H. Knox, K. D. Kroeger, J. McCombs, J. P. Megonigal, M. Lu, J. T. Morris, A. E. Sutton-Grier, T. G. Troxler, D. E. Weller, Uncertainty in United States coastal wetland greenhouse gas inventorying. *Environ. Res. Lett.* **13**, 115005 (2018).

9. A. N. Al-Haj, R. W. Fulweiler, A synthesis of methane emissions from shallow vegetated coastal ecosystems. *Glob. Change Biol.* **26**, 2988–3005 (2020).

10. M. Schuerch, T. Spencer, S. Temmerman, M. L. Kirwan, C. Wolff, D. Lincke, C. J. McOwen, M. D. Pickering, R. Reef, A. T. Vafeidis, J. Hinkel, R. J. Nicholls, S. Brown, Future response of global coastal wetlands to sea-level rise. *Nature* **561**, 231–234 (2018).

11. P. I. Macreadie, A. Anton, J. A. Raven, N. Beaumont, R. M. Connolly, D. A. Friess, J. J. Kelleway, H. Kennedy, T. Kuwae, P. S. Lavery, C. E. Lovelock, D. A. Smale, E. T. Apostolaki, T. B. Atwood, J. Baldock, T. S. Bianchi, G. L. Chmura, B. D. Eyre, J. W. Fourqurean, J. M. Hall-Spencer, M. Huxham, I. E. Hendriks, D. Krause-Jensen, D. Laffoley, T. Luisetti, N. Marbà, P. Masque, K. J. McGlathery, J. P. Megonigal, D. Murdiyarso, B. D. Russell, R. Santos, O. Serrano, B. R. Silliman, K. Watanabe, C. M. Duarte, The future of blue carbon science. *Nat. Commun.* **10**, 3998 (2019).

12. N. Saintilan, B. Horton, T. E. Törnqvist, E. L. Ashe, N. S. Khan, M. Schuerch, C. Perry, R. E. Kopp, G. G. Garner, N. Murray, K. Rogers, S. Albert, J. Kelleway, T. A. Shaw, C. D. Woodroffe, C. E. Lovelock, M. M. Goddard, L. B. Hutley, K. Kovalenko, L. Feher, G. Guntenspergen, Widespread retreat of coastal habitat is likely at warming levels above 1.5°C. *Nature* **621**, 112–119 (2023).

13. J. A. Langley, K. L. McKee, D. R. Cahoon, J. A. Cherry, J. P. Megonigal, Elevated CO_2 stimulates marsh elevation gain, counterbalancing sea-level rise. *Proc. Natl. Acad. Sci. U.S.A.* **106**, 6182–6186 (2009).

14. P. Mueller, T. J. Mozdzer, J. A. Langley, L. R. Aoki, G. L. Noyce, J. P. Megonigal, Plant species determine tidal wetland methane response to sea level rise. *Nat. Commun.* **11**, 5154 (2020).

15. G. L. Noyce, J. P. Megonigal, Biogeochemical and plant trait mechanisms drive enhanced methane emissions in response to whole-ecosystem warming. *Biogeosciences* **18**, 2449–2463 (2021).

16. G. L. Noyce, A. J. Smith, M. L. Kirwan, R. L. Rich, J. P. Megonigal, Oxygen priming induced by elevated CO_2 reduces carbon accumulation and methane emissions in coastal wetlands. *Nat. Geosci.* **16**, 63–68 (2023).

17. N. Serrano-Silva, Y. Sarria-Guzmán, L. Dendooven, M. Luna-Guido, Methanogenesis and methanotrophy in soil: A review. *Pedosphere* **24**, 291–307 (2014).

18. J. Le Mer, P. Roger, Production, oxidation, emission and consumption of methane by soils: A review. *Eur. J. Soil Biol.* **37**, 25–50 (2001).

19. J. P. Megonigal, W. H. Schlesinger, Methane-limited methanotrophy in tidal freshwater swamps. *Global Biogeochem. Cycles* **16**, 35-1–35-10 (2002).

20. S. Guerrero-Cruz, A. Vaksmaa, M. A. Horn, H. Niemann, M. Pijuan, A. Ho, Methanotrophs: Discoveries, environmental relevance, and a perspective on current and future applications. *Front. Microbiol.* **12**, 678057 (2021).

21. Y. Gao, Y. Wang, H.-S. Lee, P. Jin, Significance of anaerobic oxidation of methane (AOM) in mitigating methane emission from major natural and anthropogenic sources: A review of AOM rates in recent publications. *Environ. Sci. Adv.* **1**, 401–425 (2022).

22. K. Knittel, A. Boetius, Anaerobic oxidation of methane: Progress with an unknown process. *Annu. Rev. Microbiol.* **63**, 311–334 (2009).

23. R. J. W. Meulepas, C. G. Jagersma, J. Gieteling, C. J. N. Buisman, A. J. M. Stams, P. N. L. Lens, Enrichment of anaerobic methanotrophs in sulfate-reducing membrane bioreactors. *Biotechnol. Bioeng.* **104**, 458–470 (2009).

24. W. La, X. Han, C.-Q. Liu, H. Ding, M. Liu, F. Sun, S. Li, Y. Lang, Sulfate concentrations affect sulfate reduction pathways and methane consumption in coastal wetlands. *Water Res.* **217**, 118441 (2022).

25. A. Mostovaya, M. Wind-Hansen, P. Rousteau, L. A. Bristow, B. Thamdrup, Sulfate- and iron-dependent anaerobic methane oxidation occurring side-by-side in freshwater lake sediment. *Limnol. Oceanogr.* **67**, 231–246 (2022).

26. K. F. Ettwig, M. K. Butler, D. Le Paslier, E. Pelletier, S. Mangenot, M. M. M. Kuypers, F. Schreiber, B. E. Dutilh, J. Zedelius, D. De Beer, J. Gloerich, H. J. C. T. Wessels, T. Van Alen, F. Luesken, M. L. Wu, K. T. Van De Pas-Schoonen, H. J. M. Op Den Camp, E. M. Janssen-Megens, K. J. Francoijs, H. Stunnenberg, J. Weissenbach, M. S. M. Jetten, M. Strous, Nitrite-driven anaerobic methane oxidation by oxygenic bacteria. *Nature* **464**, 543–548 (2010).

27. A. A. Raghoebarsing, A. Pol, K. T. Van De Pas-Schoonen, A. J. P. Smolders, K. F. Ettwig, W. I. C. Rijpstra, S. Schouten, J. S. Sinninghe Damsté, H. J. M. Op Den Camp, M. S. M. Jetten, M. Strous, A microbial consortium couples anaerobic methane oxidation to denitrification. *Nature* **440**, 918–921 (2006).

28. B. L. Hu, L. D. Shen, X. Lian, Q. Zhu, S. Liu, Q. Huang, Z. F. He, S. Geng, D. Q. Cheng, L. P. Lou, X. Y. Xu, P. Zheng, Y. F. He, Evidence for nitrite-dependent anaerobic methane oxidation as a previously overlooked microbial methane sink in wetlands. *Proc. Natl. Acad. Sci. U.S.A.* **111**, 4495–4500 (2014).

29. J. Wang, C. Cai, Y. Li, M. Hua, J. Wang, H. Yang, P. Zheng, B. Hu, Denitrifying anaerobic methane oxidation: A previously overlooked methane sink in intertidal zone. *Environ. Sci. Technol.* **53**, 203–212 (2019).

30. E. E. Rios-Del Toro, E. I. Valenzuela, N. E. López-Lozano, M. G. Cortés-Martínez, M. A. Sánchez-Rodríguez, O. Calvario-Martínez, S. Sánchez-Carrillo, F. J. Cervantes, Anaerobic ammonium oxidation linked to sulfate and ferric iron reduction fuels nitrogen loss in marine sediments. *Biodegradation* **29**, 429–442 (2018).

31. M. F. Haroon, S. Hu, Y. Shi, M. Imelfort, J. Keller, P. Hugenholtz, Z. Yuan, G. W. Tyson, Anaerobic oxidation of methane coupled to nitrate reduction in a novel archaeal lineage. *Nature* **500**, 567–570 (2013).

32. K. E. A. Segarra, C. Comerford, J. Slaughter, S. B. Joye, Impact of electron acceptor availability on the anaerobic oxidation of methane in coastal freshwater and brackish wetland sediments. *Geochim. Cosmochim. Acta* **115**, 15–30 (2013).

33. K. F. Ettwig, S. Shima, K. T. van de Pas-Schoonen, J. Kahnt, M. H. Medema, H. J. M. Op den Camp, M. S. M. Jetten, M. Strous, Denitrifying bacteria anaerobically oxidize methane in the absence of archaea. *Environ. Microbiol.* **10**, 3164–3173 (2008).

34. K. F. Ettwig, T. van Alen, K. T. van de Pas-Schoonen, M. S. M. Jetten, M. Strous, Enrichment and molecular detection of denitrifying methanotrophic bacteria of the NC10 phylum. *Appl. Environ. Microbiol.* **75**, 3656–3662 (2009).

35. L. Shen, H. Wu, X. Liu, J. Li, Cooccurrence and potential role of nitrite- and nitrate-dependent methanotrophs in freshwater marsh sediments. *Water Res.* **123**, 162–172 (2017).

36. J. Wang, M. Hua, C. Cai, J. Hu, J. Wang, H. Yang, F. Ma, H. Qian, P. Zheng, B. Hua, Spatial-temporal pattern of sulfate-dependent anaerobic methane oxidation in an intertidal zone of the East China Sea. *Appl. Environ. Microbiol.* **85**, e02638-18 (2019).

37. F. Chen, Y. Zheng, L. Hou, J. Zhou, G. Yin, M. Liu, Denitrifying anaerobic methane oxidation in marsh sediments of Chongming eastern intertidal flat. *Mar. Pollut. Bull.* **150**, 110681 (2020).

38. F. Chen, Y. Zheng, L. Hou, Y. Niu, D. Gao, Z. An, J. Zhou, G. Yin, H. Dong, P. Han, X. Liang, M. Liu, Microbial abundance and activity of nitrite/nitrate-dependent anaerobic methane oxidizers in estuarine and intertidal wetlands: Heterogeneity and driving factors. *Water Res.* **190**, 116737 (2021).

39. Y. Zheng, L. Hou, F. Chen, J. Zhou, M. Liu, G. Yin, J. Gao, P. Han, Denitrifying anaerobic methane oxidation in intertidal marsh soils: Occurrence and environmental significance. *Geoderma* **357**, 113943 (2020).

40. S. Song, C. Zhang, Y. Gao, X. Zhu, R. Wang, M. Wang, Y. Zheng, L. Hou, M. Liu, D. Wu, Responses of wetland soil bacterial community and edaphic factors to two-year experimental

warming and *Spartina alterniflora* invasion in Chongming Island. *J. Clean. Prod.* **250**, 119502 (2020).

41. Y. Liu, X. Liu, K. Cheng, L. Li, X. Zhang, J. Zheng, J. Zheng, G. Pan, Responses of methanogenic and methanotrophic communities to elevated atmospheric CO_2 and temperature in a paddy field. *Front. Microbiol.* **7**, 1895 (2016).

42. Y. Zhu, K. J. Purdy, Ö. Eyice, L. Shen, S. F. Harpenslager, G. Yvon-Durocher, A. J. Dumbrell, M. Trimmer, Disproportionate increase in freshwater methane emissions induced by experimental warming. *Nat. Clim. Change* **10**, 685–690 (2020).

43. Y. Wang, Z. Hu, L. Shen, C. Liu, A. R. M. T. Islam, Z. Wu, H. Dang, S. Chen, The process of methanogenesis in paddy fields under different elevated CO_2 concentrations. *Sci. Total Environ.* **773**, 145629 (2021).

44. N. D. Ward, J. P. Megonigal, B. Bond-Lamberty, V. L. Bailey, D. Butman, E. A. Canuel, H. Diefenderfer, N. K. Ganju, M. A. Goñi, E. B. Graham, C. S. Hopkinson, T. Khangaonkar, J. A. Langley, N. G. McDowell, A. N. Myers-Pigg, R. B. Neumann, C. L. Osburn, R. M. Price, J. Rowland, A. Sengupta, M. Simard, P. E. Thornton, M. Tzortziou, R. Vargas, P. B. Weisenhorn, L. Windham-Myers, Representing the function and sensitivity of coastal interfaces in Earth system models. *Nat. Commun.* **11**, 2458 (2020).

45. G. L. Noyce, M. L. Kirwan, R. L. Rich, J. P. Megonigal, Asynchronous nitrogen supply and demand produce nonlinear plant allocation responses to warming and elevated CO_2. *Proc. Natl. Acad. Sci. U.S.A.* **116**, 21623–21628 (2019).

46. G. A. Miley, R. P. Kiene, Sulfate reduction and porewater chemistry in a gulf coast*Juncus roemerianus* (Needlerush) marsh. *Estuaries* **27**, 472–481 (2004).

47. A. Robador, V. Brüchert, B. B. Jørgensen, The impact of temperature change on the activity and community composition of sulfate-reducing bacteria in arctic versus temperate marine sediments. *Environ. Microbiol.* **11**, 1692–1703 (2009).

48. L. Seidel, V. Sachpazidou, M. Ketzer, S. Hylander, A. Forsman, M. Dopson, Long-term warming modulates diversity, vertical structuring of microbial communities, and sulfate reduction in coastal Baltic Sea sediments. *Front. Microbiol.* **14**, 1099445 (2023).

49. A. L. Bullock, A. E. Sutton-Grier, J. P. Megonigal, Anaerobic metabolism in tidal freshwater wetlands: III. Temperature regulation of iron cycling. *Estuar. Coast.* **36**, 482–490 (2013).

50. J. A. Langley, T. J. Mozdzer, K. A. Shepard, S. B. Hagerty, J. P. Megonigal, Tidal marsh plant responses to elevated CO_2, nitrogen fertilization, and sea level rise. *Glob. Change Biol.* **19**, 1495–1503 (2013).

51. M. Nie, M. Lu, J. Bell, S. Raut, E. Pendall, Altered root traits due to elevated CO_2: A meta-analysis. *Glob. Ecol. Biogeogr.* **22**, 1095–1105 (2013).

52. W. E. Connell, W. H. Patrick, Sulfate reduction in soil: Effects of redox potential and *p*H. *Science* **159**, 86–87 (1968).

53. J. P. Megonigal, J. R. Holmquist, Dataset: 1999 CO2xCommunity experiment belowground biomass, Smithsonian Environmental Research Center (2021); https://doi.org/10.25573/serc.13073249.

54. E. J. Beal, C. H. House, V. J. Orphan, Manganese- and iron-dependent marine methane oxidation. *Science* **325**, 184–187 (2009).

55. Y. Zhang, X. Zhang, F. Wang, W. Xia, Z. Jia, Exogenous nitrogen addition inhibits sulfate-mediated anaerobic oxidation of methane in estuarine coastal sediments. *Ecol. Eng.* **158**, 106021 (2020).

56. Z. Wang, J. Li, X. Xu, K. Li, Q. Chen, Denitrifying anaerobic methane oxidation and mechanisms influencing it in Yellow River Delta coastal wetland soil, China. *Chemosphere* **298**, 134345 (2022).

57. X. Liu, A. H. W. Beusen, L. P. H. Van Beek, J. M. Mogollón, X. Ran, A. F. Bouwman, Exploring spatiotemporal changes of the Yangtze River (Changjiang) nitrogen and phosphorus

sources, retention and export to the East China Sea and Yellow Sea. *Water Res.* **142**, 246–255 (2018).

58. J. Wang, A. H. W. Beusen, X. Liu, R. Van Dingenen, F. Dentener, Q. Yao, B. Xu, X. Ran, Z. Yu, A. F. Bouwman, Spatially explicit inventory of sources of nitrogen inputs to the Yellow Sea, East China Sea, and South China Sea for the period 1970–2010. *Earth's Future* **8**, e2020EF001516 (2020).

59. A. H. W. Beusen, J. C. Doelman, L. P. H. Van Beek, P. J. T. M. Van Puijenbroek, J. M. Mogollón, H. J. M. Van Grinsven, E. Stehfest, D. P. Van Vuuren, A. F. Bouwman, Exploring river nitrogen and phosphorus loading and export to global coastal waters in the shared socio-economic pathways. *Glob. Environ. Change* **72**, 102426 (2022).

60. D. J. Velinsky, B. Paudel, T. Quirk, M. Piehler, A. Smyth, Salt marsh denitrification provides a significant nitrogen sink in Barnegat Bay, New Jersey. *J. Coast. Res.* **78**, 70–78 (2017).

61. T. E. Jordan, D. L. Correll, Continuous automated sampling of tidal exchanges of nutrients by brackish marshes. *Estuar. Coast. Shelf Sci.* **32**, 527–545 (1991).

62. B. Thamdrup, H. G. R. Steinsdóttir, A. D. Bertagnolli, C. C. Padilla, N. V Patin, E. Garcia-Robledo, L. A. Bristow, F. J. Stewart, Anaerobic methane oxidation is an important sink for methane in the ocean's largest oxygen minimum zone. *Limnol. Oceanogr.* **64**, 2569–2585 (2019).

63. W. Michaelis, R. Seifert, K. Nauhaus, T. Treude, V. Thiel, M. Blumenberg, K. Knittel, A. Gieseke, K. Peterknecht, T. Pape, A. Boetius, R. Amann, B. B. Jørgensen, F. Widdel, J. Peckmann, N. V. Pimenov, M. B. Gulin, Microbial reefs in the black sea fueled by anaerobic oxidation of methane. *Science* **297**, 1013–1015 (2002).

64. A. J. M. Stams, C. M. Plugge, Electron transfer in syntrophic communities of anaerobic bacteria and archaea. *Nat. Rev. Microbiol.* **7**, 568–577 (2009).

65. G. Muyzer, A. J. M. Stams, The ecology and biotechnology of sulphate-reducing bacteria. *Nat. Rev. Microbiol.* **6**, 441–454 (2008).

66. S. Bertram, M. Blumenberg, W. Michaelis, M. Siegert, M. Krüger, R. Seifert, Methanogenic capabilities of ANME-archaea deduced from ^{13}C-labelling approaches. *Environ. Microbiol.* **15**, 2384–2393 (2013).

67. S. C. Neubauer, J. P. Megonigal, Moving beyond global warming potentials to quantify the climatic role of ecosystems. *Ecosystems* **18**, 1000–1013 (2015).

68. M. A. Waqas, Y. Li, M. N. Ashraf, W. Ahmed, B. Wang, M. F. Sardar, P. Ma, R. Li, Y. Wan, Y. Kuzyakov, Long-term warming and elevated CO_2 increase ammonia-oxidizing microbial communities and accelerate nitrification in paddy soil. *Appl. Soil Ecol.* **166**, 104063 (2021).

69. E. E. Roden, R. G. Wetzel, Organic carbon oxidation and suppression of methane production by microbial Fe(III) oxide reduction in vegetated and unvegetated freshwater wetland sediments. *Limnol. Oceanogr.* **41**, 1733–1748 (1996).

70. J. V. Weiss, D. Emerson, J. P. Megonigal, Geochemical control of microbial Fe(III) reduction potential in wetlands: Comparison of the rhizosphere to non-rhizosphere soil. *FEMS Microbiol. Ecol.* **48**, 89–100 (2004).

71. R. L. Rich, A. Stefanski, R. A. Montgomery, S. E. Hobbie, B. A. Kimball, P. B. Reich, Design and performance of combined infrared canopy and belowground warming in the B4WarmED (Boreal Forest Warming at an Ecotone in Danger) experiment. *Glob. Change Biol.* **21**, 2334–2348 (2015).

72. J. K. Keller, A. A. Wolf, P. B. Weisenhorn, B. G. Drake, J. P. Megonigal, Elevated CO_2 affects porewater chemistry in a brackish marsh. *Biogeochemistry* **96**, 101–117 (2009).

73. V. Amrhein, S. Greenland, B. McShane, Scientists rise up against statistical significance. *Nature* **567**, 305–307 (2019).

74. K. G. Lloyd, M. J. Alperin, A. Teske, Environmental evidence for net methane production and oxidation in putative anerobic methanotrophic (ANME) archaea. *Environ. Microbiol.* **13**, 2548–2564 (2011).

75. A. Vigneron, P. Cruaud, P. Pignet, J.-C. Caprais, M.-A. Cambon-Bonavita, A. Godfroy, L. Toffin, Toffin, archaeal and anaerobic methane oxidizer communities in the Sonora Margin cold seeps, Guaymas Basin (Gulf of California). *ISME J.* **7**, 1595–1608 (2013).
